# FAST-SeqS: A Simple and Efficient Method for the Detection of Aneuploidy by Massively Parallel Sequencing

**DOI:** 10.1371/journal.pone.0041162

**Published:** 2012-07-18

**Authors:** Isaac Kinde, Nickolas Papadopoulos, Kenneth W. Kinzler, Bert Vogelstein

**Affiliations:** The Ludwig Center for Cancer Genetics and Therapeutics and The Howard Hughes Medical Institute, Johns Hopkins Kimmel Cancer Center, Baltimore, Maryland, United States of America; Institut Jacques Monod, France

## Abstract

Massively parallel sequencing of cell-free, maternal plasma DNA was recently demonstrated to be a safe and effective screening method for fetal chromosomal aneuploidies. Here, we report an improved sequencing method achieving significantly increased throughput and decreased cost by replacing laborious sequencing library preparation steps with PCR employing a single primer pair designed to amplify a discrete subset of repeated regions. Using this approach, samples containing as little as 4% trisomy 21 DNA could be readily distinguished from euploid samples.

## Introduction

A major chromosomal abnormality is detected in approximately 1 of 140 live births [1] and in a much higher fraction of fetuses that do not reach term or are still-born [2]. The most common aneuploidy is trisomy 21 (Down syndrome), which currently occurs in 1 of 730 births [1]. Though less common than trisomy 21, trisomy 18 (Edwards Syndrome) and trisomy 13 (Patau syndrome) occur in 1 in 5,500 and 1 in 17,200 live births, respectively [1]. A large variety of congenital defects, growth deficiencies, and intellectual disabilities are found in children with chromosomal aneuploidies, and these present life-long challenges to families and societies [3]. For these reasons, much effort has been devoted to detecting chromosome abnormalities during early fetal life, at a time when therapeutic abortions can be offered as an option to prospective parents.

There are a variety of prenatal tests that can indicate increased risk for fetal aneuploidy, although invasive diagnostic tests such as amniocentesis or chorionic villus sampling are the current gold standard [4] and are associated with a non-negligible risk of fetal loss. More reliable, non-invasive tests for fetal aneuploidy have therefore long been sought. The most promising of these are based on the detection of fetal DNA in maternal plasma, as pioneered by Lo’s group [5]. It has recently been demonstrated that massively parallel sequencing of libraries generated from maternal plasma can reliably detect chromosome 21 abnormalities [6], [7]. In the most comprehensive study to date [8], 98.6% of fetuses with trisomy 21 were detected in maternal plasma, with a false positive rate of 0.2 percent. In an additional 0.8 percent of samples, the test failed to give a result. These exciting studies promise a new era of non-invasive prenatal testing.

Currently, almost half of trisomy 21 babies are born to mothers less than 35 years of age, as more than 80% of pregnant women are under 35 [9], [10]. Though the risk of invasive procedures is thought to outweigh the benefit of invasive testing for eligible young mothers, it is clear that the vast majority of births associated with chromosomal aneuploidies could be safely identified with reliable non-invasive tests that could be administered to all pregnant women. Prenatal testing is an extraordinarily stressful exercise for pregnant mothers and their families, and the more rapid the process, the better.

To achieve this goal with circulating fetal DNA testing, decreases in cost and increases in throughput will be necessary. There are three major components of plasma DNA testing: preparation of DNA libraries for loading on the sequencing instrument, the sequencing of these libraries, and their analysis. The second component is being addressed by instrument manufacturers, who have made remarkable progress over the last few years. Potential improvements in the first and third components are the subject of the current study.

The only commercially available tests for circulating fetal DNA aneuploidy [8], [11] involve the preparation of whole genome libraries and the analysis of a sufficient number of sequences on the relevant chromosomes to reliably detect small differences in copy number. The preparation of whole genome libraries involves several sequential steps - including end-repair, 5′-phosphorlyation, addition of a terminal dA nucleotide to the 3′ ends of the fragments, ligation of the fragments to adapters, and PCR amplification of the ligated products - many of which require intervening purifications. The PCR products are then quantified and loaded on the sequencing instrument. Following the sequencing run, the tags are aligned to the human genome and assessed with Digital Karyotyping [12], i.e., the number of tags per genomic locus is used to construct a virtual karyotype. Another recently described test involves fewer, but still a large number of, steps to prepare libraries for sequencing [13], [14].

We reasoned that this process could be simplified if a defined number of fragments from throughout the genome could be amplified using a single primer pair, obviating the need for end-repair, terminal 3′-dA addition, or ligation to adapters. Furthermore, the smaller number of fragments to be assessed (compared to the whole genome) would streamline the genome matching and analysis processes. Here we detail our approach, which we have named “Fast Aneuploidy Screening Test-Sequencing System (henceforth FAST-SeqS).

## Materials and Methods

### Templates

Control DNA was obtained from normal spleen, peripheral blood white blood cells (WBCs), or plasma from patients (Table S1) giving written informed consent after approval by the institutional review board of The Johns Hopkins University. Fibroblast DNA from five individuals with trisomy 21 (NA02767, NA04616, NG05120, NG05397, and NG07438), two with trisomy 18 (NA03623 and NG12614), and one with trisomy 13 (NA03330), all with karyotype-confirmed aneuploidies, were purchased from the Coriell Institute for Medical Research (Camden, New Jersey). A total of 33 ng of DNA was used for each experiment. All templates were quantified by OD, except for the mixing experiments in which the templates were quantified by Digital PCR [15] to achieve a more accurate estimate of concentration.

### Sequencing Library Preparation

The most significant time savings in FAST-SeqS is afforded by the replacement of currently used, laborious library preparation steps with an amplification using a single primer pair designed to amplify a discrete subset of repeated regions (see ‘Results and Discussion’ section). Templates were amplified as described by Kinde *et al.* [16] in which individual template molecules are tagged with a unique identifier DNA sequence. Though the unique identifier sequences turned out to be not necessary for FAST-SeqS (see ‘Results and Discussion’ section), we included them in the initial experimental design and continued to use them once they were observed to provide robust PCR products in our initial experiments. Briefly, FAST-1 amplification primers each contained a different 5′ universal primer sequence (UPS) followed by sequences allowing amplification of well-dispersed, repeated elements (see ‘Results and Discussion’ section and Table S2). Additionally, the forward primer contained either a stretch of 16 or 20 degenerate bases immediately 3′ to its UPS (Table S2). PCR was performed using Phusion Hot Start II Polymerase (Thermo Scientific, cat. no. F-549 L) in a total of 50 µL of 1× Phusion HF buffer containing 0.5 µM each primer and two units of polymerase under the following cycling conditions: 98°C for 120 s, followed by two cycles of 98°C for 10 s, 57°C for 120 s, and 72°C for 120 s. The initial amplification primers were removed with AMPure XP beads (Beckman Coulter Genomics, cat. no. A63880) according to the manufacturer with the exception that the beads were added at only 1.4× the PCR volume and the elution volume was reduced to 10 µL of TE. The elution was used directly for a second round of amplification using primers that annealed to the UPS site introduced by the first round primers and that additionally contained the 5′ grafting sequences necessary for hybridization to the Illumina flow cell (Table S2). Further, we introduced one of five indexes (“barcodes”) (Table S2) to each sample in the reverse primer to later allow multiplexed sequencing. The second round of PCR was performed using Phusion Hot Start II Polymerase in a total of 50 µL of 1× Phusion HF buffer containing 0.5 µM each primer and two units of polymerase under the following cycling conditions: 98°C for 120 s, followed by 13 cycles of 98°C for 10 s, 65°C for 15 s, and 72°C for 15 s. Amplification products were again purified with AMPure XP beads and were quantified by spectrophotometry, real time PCR or on an Agilent 2100 Bioanalyzer; all methods of quantification yielded similar results. All oligonucleotides were purchased from IDT (Coralville, Iowa).

### Sequence Tag Filtering and Alignment

Thirty-seven base sequence tags passing the Illumina chastity filter and containing at least three correct terminal bases of the amplification primer were filtered for quality by masking any base with a quality score <20 with an N using a custom script. Thus, tags with low quality bases were given the opportunity to align by considering only their most reliable bases. After quality masking, only the distinct sequences were aligned to the human genome (hg19 [17]) using Bowtie 0.12.7 [18]. When building the reference index for Bowtie, we included unresolved or unplaced contigs [19] to ensure the most accurate alignments. Sequences that aligned uniquely with up to one mismatch (using the flags –m 1 and –v 1, respectively) were retained and their alignments were matched back to the original data. Because tag alignment to a discrete set of chromosomal positions is simpler than alignment to the entire genome, the post-sequencing analysis process was very rapid. In fact, this mapping plus subsequent statistical analysis could be completed in less than 30 min per sample with a single computer housing two six-core CPUs (Intel Xeon X5680).

### Normalization

Massively parallel sequencing will generate a different number of sequence tags from each sample, as well as from different sequencing runs of the same sample, due to stochastic and experimental variations. Thus, it is essential to normalize the data to make meaningful comparisons of the type used here. Although it would be most straightforward to simply express tag counts as a fraction of the total number of tags sequenced in an experiment, this normalization is too simplistic and is highly susceptible to systemic biases that frequently plague next generation sequencing of both DNA and RNA templates. For example, normalization for local GC content is routinely used in Digital Karyotyping [12] analyses such as that used for the diagnosis of trisomy 21 [8], [20].

**Figure 1:**
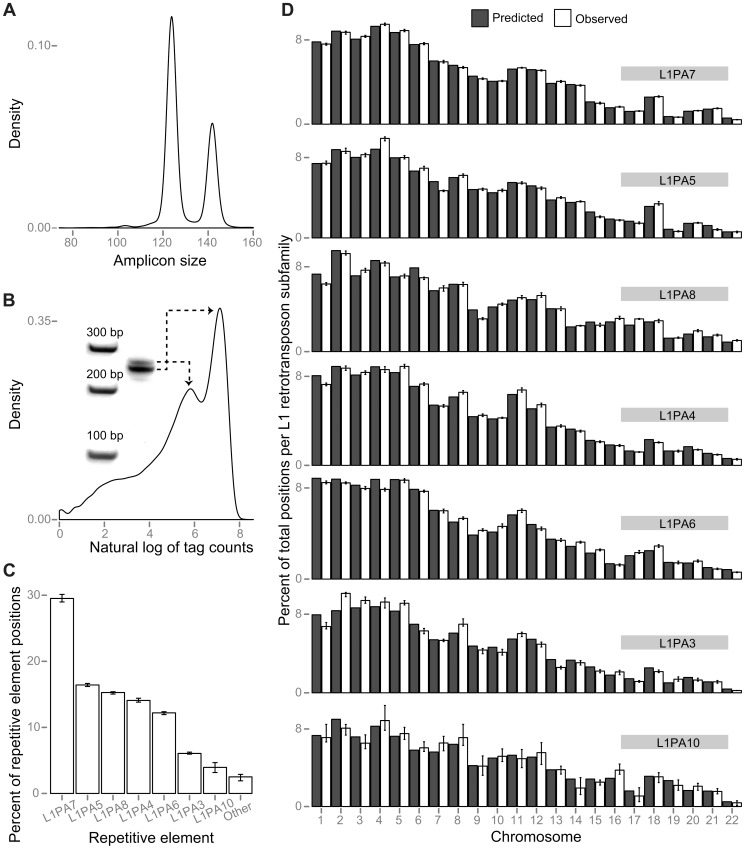
Comparison of observed and predicted distributions of FAST-SeqS amplification products. (A) A density plot of the expected distribution of fragment lengths, with peaks at 124 and 142 bp. (B) A density plot of the actual tag counts obtained in eight normal plasma DNAs. The 124 bp fragments are preferentially amplified compared to the 142 bp fragments, likely due to an amplification bias towards smaller fragments. Inset: polyacrylamide gel of a representative FAST-SeqS sequencing library. Note: the amplification products contain an additional ∼120 bp of flanking sequence to facilitate sequencing (Table S2). (C) The average representation of the most frequently observed L1 retrotransposon subfamilies in eight normal plasma samples. Roughly 97% of uniquely aligning tags arise from positions representing only seven L1 retrotransposon subfamilies. (D) A detailed examination of the average number of observed positions per chromosome from eight normal plasma DNAs compared with the number predicted by RepeatMasker for each of the seven L1 retrotransposon subfamilies noted in (C). Error bars in each panel depict the range.

Because of the bimodal size distribution of the amplicons obtained with the FAST-1 primer pair (see ‘Results and Discussion’ section), we predicted that the majority of bias in FAST-1 amplifications would be due to the potential over-representation of the smaller-sized fragments. This bias could either be introduced during library preparation or during solid-phase bridge PCR on the Illumina flow cell. We found that an appropriate normalization for this distribution could be obtained using the quantile method [21], used extensively within the microarray community. By organizing our data into a list of positions (equivalent to probes in microarray data), each associated with a tag count (equivalent to intensities in microarray data), we were able to apply standard quantile normalization to FAST-SeqS data. To best approximate the microarray data format, we chose to only analyze positions that were shared within each experimental group (e.g., the data from eight normal plasma samples). As the FAST-1 primers amplified a highly reproducible set of positions, this generally only eliminated <1% of the data. To maximize reproducibility, we excluded positions aligning to unresolved or unplaced contigs and those aligning to sex chromosomes, although inclusion of these chromosomes only marginally increased variability between experiments (e.g., in eight normal plasma samples, the maximum z-score from any chromosome rose from 1.9 to 2.3). The inclusion of sex chromosomes could be useful for other applications, such as detecting aneuploidies involving chromosome X or determining the gender of a sample (i.e., by the presence or absence of sequences aligning to chromosome Y).

We implemented the quantile normalization [21] for each experimental group (each of which contained multiple samples; Table S3) by performing the following steps:

1. generating a table of tag counts for each sample where each row represents a unique position (note that all tables will be of equal length as only the shared positions in each experiment were analyzed);
2. sorting the rows in each table based on tag counts, resulting in each table having a different order of positions;
3. determining the mean tag count for each row across all samples;
4. replacing an individual sample’s tag count with the mean tag count for all samples at each row; and
5. sorting the tag counts for each sample’s table back to their original order based on position.

The raw distribution of our data was always negatively skewed (see ‘Results and Discussion’ section). We excluded the positions falling within the left tail of each experiment’s distribution (the positions containing the smallest number of tags) from our analysis by:

1. estimating the distribution of normalized values (see ‘Results and Discussion’ section);
2. determining the inflection point between the two peaks of the bimodal distribution; and
3. considering the positions that had a relative density below the inflection point as the left tail.

Once the left tail was determined and positions within it discarded, the quantile normalization was repeated. Through this process, each sample within an experimental group had the same sum total of tags and an identical distribution of counts, so direct comparisons could be made. We automated the quantile normalization in R [22]. The entire normalization procedure routinely took less than a few minutes to complete.

**Figure 2:**
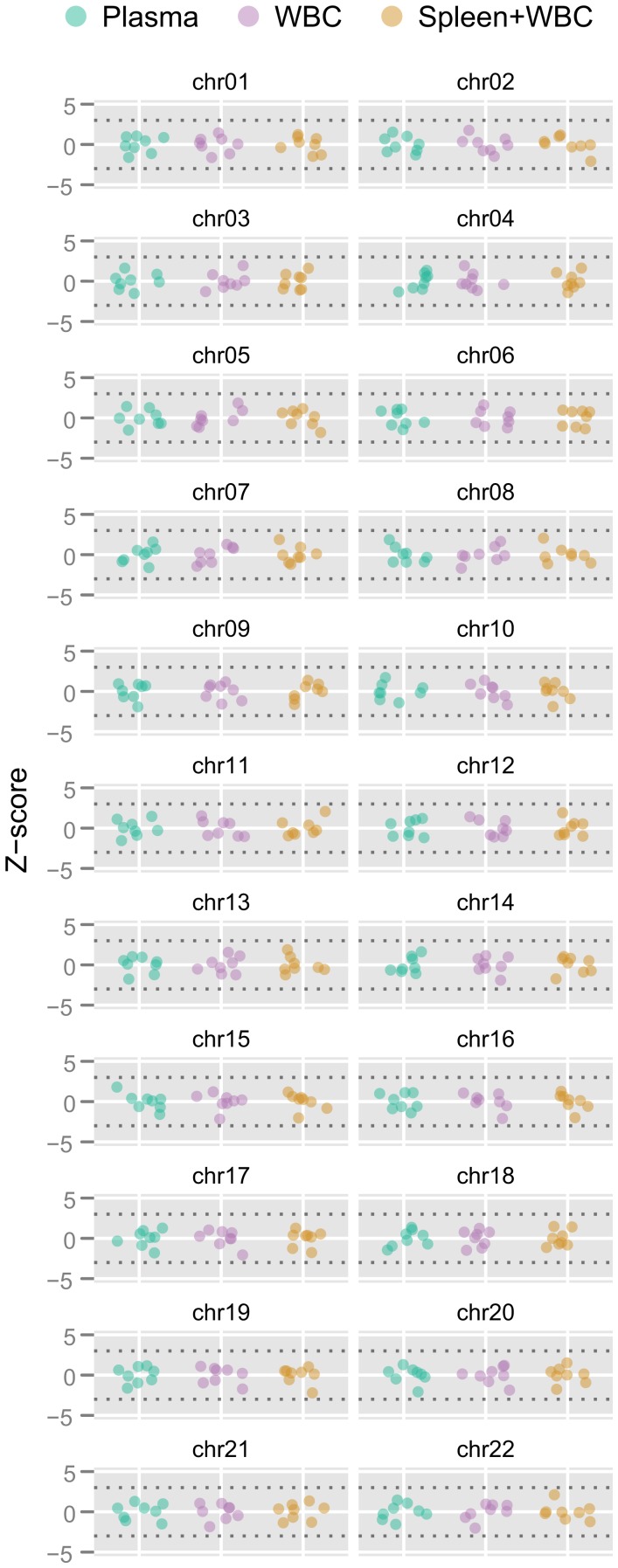
Demonstration of FAST-SeqS reproducibility among different samples, sequencing instruments, and sequencing depth. FAST-SeqS was performed on eight normal plasma DNA samples, their corresponding matched peripheral blood white blood cell (WBC) DNA, and on the splenic or WBC DNA of an additional eight unrelated individuals. The eight samples within each experiment constituted the reference group (see ‘Materials and Methods’ section) from which the plotted z-scores were calculated. No autosome in any sample had a z-score outside the range of −3.0 and 3.0 (dotted lines). Despite 3-fold less sequencing of the splenic or WBC samples, the z-scores (range: −2.2 to 2.1) were similar to those obtained from the plasma (range: −2.1 to 1.9) and matched WBC DNA samples (range: −2.2 to 1.9).

**Figure 3:**
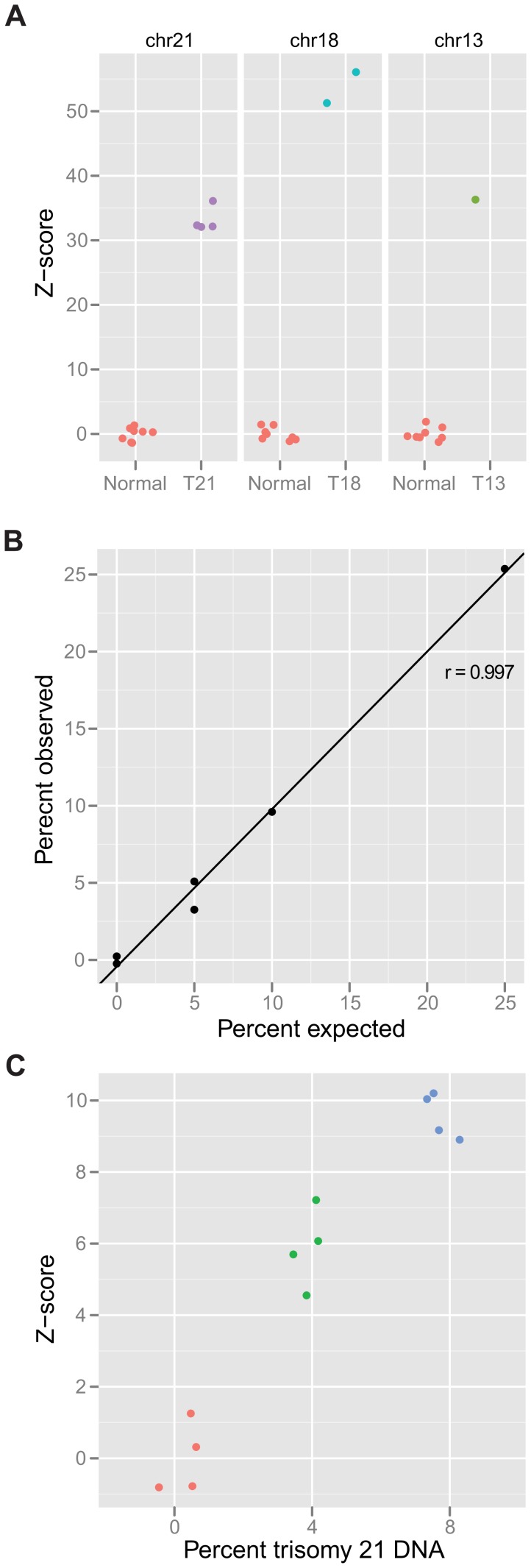
Accurate discrimination of euploid DNA samples from those containing trisomic DNA. (A) Comparison of z-scores from patients with trisomy 21 (n = 4), trisomy 18 (n = 2), and trisomy 13 (n = 1) with eight normal spleen or peripheral blood white blood cell (WBC) DNAs. The z-scores displayed represent the relevant chromosome for the comparison. The maximum z-score observed for any of the compared normal chromosomes was 1.9 (chr13). (B) Control WBC DNA was analyzed alone (n = 2) or when mixed with DNA from a patient with trisomy 21 at 5% (n = 2), 10% (n = 1), or 25% (n = 1) levels. A tight correlation existed between the expected and observed fractions of extra chromosome 21 (r = 0.997 by Pearson correlation test, n = 6). (C) Control WBC DNA was analyzed alone (z-score range: −0.8 to 1.3) or when mixed with DNA from a patient with trisomy 21 at 4% (z-score range: 4.5 to 7.2) or 8% (z-score range: 8.9 to 10.) levels. Each experiment in (C) was performed in quadruplicate.

### Quantitative Determination of Aneuploidy Status

A common method of determining the aneuploidy status of a particular sample in Digital Karyotyping-based [12] assays is by comparison of z-scores [6], [11], [23], [24]. Through this method, one determines the mean and standard deviation of tag counts lying within a chromosome of interest in a group of reference samples (e.g., samples with known euploid content), and then creates a standardized score (i.e., z-score) for a chromosome of interest for each sample as follows:
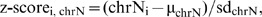
where i represents the sample to be standardized, chrN represents the normalized tag count of the sample’s chromosome, and µ_chrN_ and sd_chrN_ represent the mean and standard deviation of the normalized tag counts, respectively, of chrN in the reference group. When all samples are standardized in this way, outliers are easily detected because they have a z-score >3.0. This indicates that the normalized tag count of the outlier exceeds the mean of the reference group by at least three standard deviations.

## Results and Discussion

### Primer Selection and *in silico* Analysis

The key innovation behind FAST-SeqS, which increases throughput and lowers cost compared to traditional whole-genome sequencing fetal aneuploidy screening tests, is the use of specific primers that anneal to a subset of repeated regions dispersed throughout the genome. For maximum utility, we sought to target regions with enough similarity so that they could be amplified with a single pair of primers, but sufficiently unique to allow most of the amplified loci to be distinguished.

We began by searching a ∼6.8 Mb region of chromosome 21 (hg19 [17] coordinates 35,888,786 to 42,648,523), containing the Down syndrome critical region [25], for sequence blocks of ∼150 bp that were similar but not identical to those present on all chromosomes. To identify such blocks, we queried sequences obtained from 150 bp sliding windows incremented by 50 bp (135,186 sequences of 150 bp in length) with the BLAST-Like Alignment Tool (BLAT) algorithm [26]. We also required that the queried sequence be similar to at least three other blocks on chromosome 21, in addition to the one within the ∼6.8 Mb region described above.

Out of the 135,186 queried blocks, we found only 56 that met the following criteria:

1. contained at least 11 variant bases from the query sequence, to aid in distinguishing amplified loci;
2. contained no more than 30 variant bases from the query sequence, to increase the chance of uniform amplification; and
3. spanned no more than a total of 180 bases, to be compatible with the analysis of degraded DNA [8].

We then manually scanned the BLAT alignments of these 56 blocks to search for those that had highly similar 5′ and 3′ ends. At least three of the 56 sequences met our criteria and we designed primers for them. *In Silico* PCR [27] predicted that each primer pair would amplify many distinct sequences from every nuclear chromosome.

Sequences that were too similar could pose a problem during alignment because of the inevitable errors introduced during library preparation or sequencing. We therefore wrote a custom script to assess how many distinct sequences would remain after allowing one, two, or three errors in each ∼150 bp sequence. The theoretical amplification products of one primer pair (FAST-1) greatly outperformed the other two, and the superiority of FAST-1 was confirmed in pilot sequencing experiments.

The FAST-1 primer pair was predicted to amplify subfamilies of long interspersed nucleotide element-1 (L1 retrotransposons) in a primarily bimodal distribution of amplicon sizes, with the majority of amplicons having an average size of 124 or 142 bp (Fig. 1A). L1 retrotransposons, like other human repeats, have spread throughout the genome via retrotransposition, particularly in AT-rich regions [28]. As it is generally more difficult to uniformly amplify and sequence regions that vary widely in their GC content [8], [20], we expected that this differential localization would work in our favor.

### FAST-SeqS Yields a Highly Reproducible Subset of Sequences

An average of 38% of tags across all samples could be uniquely assigned to a genomic position (range: 31% to 45%; Table S3). As opposed to traditional whole genome amplification libraries, where the vast majority of tags align to the genome in unique positions and thus requiring that each tag be independently aligned, FAST-SeqS yielded sequences that aligned to an average of only 21,676 positions (Table S3). The number of positions to which the sequences aligned varied little compared to the range of sequence data obtained across all experiments. Though the number of uniquely aligned tags per experiment spanned a 12-fold range (1,343,382 to 16,015,347) the number of positions varied only by 0.25-fold (range: 18,484 to 24,562 positions; Table S3). Not only was the number of aligned positions similar among samples, but the identities of the positions were also remarkably similar: among samples within an experimental group, <1% of aligned tags were eliminated when limiting the analysis to positions shared among each sample.

### The Distribution of Sequenced Fragments Agrees with *in silico* Predictions

Though we only sequenced 37 bases, we could estimate the relative size of the original PCR fragment and its unique position in the genome after alignment. This exercise provided additional evidence that the actual amplification products matched those that were predicted and alerted us to a preferential amplification bias towards sequences arising from smaller fragments.

We transformed the tag counts per uniquely aligned position to a log scale – a transformation frequently performed to this class of data to induce symmetry [29] – for each group of experiments (Table S3). Next, we used a nonparametric method to estimate a smoothened distribution (a kernel density estimator, implemented in R [22] using the density function), which made it straightforward to visualize the modality of our data. After plotting the distribution using ggplot2 [30] (an R [22] package), we observed that each group of experiments showed a similar clustering of tag counts per position, consistent with a primarily bimodal distribution with a negative skew. Tags originating from smaller PCR fragments were observed to have higher average tag counts, likely due to amplification biases. A representative plot is displayed in Figure 1B.

### Autosomal Representation after Performing FAST-SeqS is Highly Reproducible

As an initial test of the performance of FAST-SeqS, we examined the representation of each autosome from different biologic sources (Table S1) using different sequencing instruments and depth (Table S3).

We first examined the representation of each autosome in the plasma DNA of seven normal females, including one biologic replicate (a total of eight samples), using only 37 cycles of sequencing in one-quarter of a lane on an Illumina HiSeq 2000 instrument. We recovered an average of 31,547,988 high quality tags per individual (range: 27,179,424 to 36,048,017 tags; Table S3). An average of 35% of these tags (range: 31 to 37%) could be uniquely mapped to one of an average of 23,681 unique chromosomal positions (range: 22,589 to 24,562 positions) when allowing up to one mismatch during alignment to hg19 [17] using Bowtie [18]. Of the uniquely aligned tags, 99.1% aligned to positions predicted to be repetitive DNA by RepeatMasker (http://www.repeatmasker.org), 97.5% of which fell into just seven L1 retrotransposon subfamilies (Fig. 1C). Additionally, as depicted in Fig. 1D, the distribution of each subfamily was not statistically distinguishable from that predicted by RepeatMasker (p = 1 for each of the seven L1 retrotransposon subfamilies when comparing the observed mean percentage of positions per chromosome with the predicted number; correlated two-tailed *t*-test).

Most importantly, the relative fraction of tags mapping to each chromosome was remarkably similar among the individual samples after normalizing [21] to compare chromosome tag counts among different samples (see ‘Materials and Methods’ section). In particular, the fraction of tags that matched to any of the autosomes in any of the eight samples studied never deviated from the average by a z-score >3.0 (Fig. 2). Of particular note, the maximum z-scores observed among the eight samples for chromosomes 21, 18, and 13 were 1.3, 1.4, and 1.0, respectively.

In the next experiment, we analyzed DNA from peripheral blood white blood cells (WBCs) from the same seven individuals who contributed plasma, including the biologic replicate (eight total samples). Four samples were sequenced on a single lane of an Illumina HiSeq 2000, yielding a mean of 10,835,559 uniquely aligned tags per sample (range: 4,905,067 to 16,015,347 tags). The maximum z-scores for any of the samples were 1.0, 1.2, and 1.6 for chromosomes 21, 18, and 13, respectively (Fig. 2).

Finally, we analyzed splenic or WBC DNA from an additional eight individuals using one-half of a lane of an Illumina GA IIx instrument, designed to yield fewer tags per sample than achieved above. Despite almost 3-fold less sequencing (average of 4,013,951 uniquely aligned tags per sample), the maximum z-scores among the samples were still only 1.3, 1.5, and 1.9 for chromosomes 21, 18, and 13, respectively, well below the widely used cutoff of 3.0 (Fig. 2).

### FAST-SeqS Readily Identifies Samples Containing Trisomic DNA, Even when Present in Low Proportions

Given the tight distributions of tags evident in Figure 2, we expected it would be straightforward to distinguish the DNA of patients with trisomies from those of normal individuals with euploid chromosome constitutions. The data depicted in Figure 3A demonstrate that this expectation was realized in each of four patients with trisomy 21. The z-scores among these trisomy 21 patients ranged from 32 to 36, while the maximum z-score among eight normal individuals was 1.3. Similarly, the z-scores of DNA from two patients with trisomy 18 and one from trisomy 13 were 51, 56, and 36, respectively, far exceeding the maximum z-scores for these chromosomes in normal individuals (Fig. 3A).

Fetal DNA accounts for a geometric mean of 13.4% of maternal DNA, depending largely on maternal weight rather than gestational age [8]. To investigate whether FAST-SeqS could distinguish samples that contained mixtures of disomic and trisomic DNA, we performed mixing experiments using DNA from patients with trisomy 21 and normal individuals. In a first experiment of this type, we mixed 5% (n = 2), 10% (n = 1), and 25% (n = 1) trisomy 21 DNA into normal WBC DNA alongside two controls (Fig. 3B), and found a tight correlation between the expected and observed fractions of extra chromosome 21 (r = 0.997 by Pearson correlation test, n = 6). In a second experiment, we evaluated mixtures that contained 4% or 8% trisomy 21 DNA. As shown in Figure 3C, there was a clear distinction between the samples containing 4% trisomy 21 DNA vs. those from normal individuals (p = 2×10^−4^ as determined by uncorrelated two-tailed *t*-test, n = 4 in each group). The samples containing 8% trisomy 21 DNA were of course even more easily distinguishable (p = 4×10^−6^ when compared to the euploid group and p = 1×10^−3^ when compared to the 4% trisomy 21 samples, both by uncorrelated two-tailed *t*-test with n = 4 for each group).

### Precise Template Counting

Finally, we evaluated whether precisely counting template molecules could further increase reproducibility. By incorporating degenerate bases at the 5′ end of one of the two FAST-1 primers (Table S2), it is possible to uniquely identify each template molecule giving rise to a PCR product [16]. This could potentially increase accuracy by minimizing the chance that the same template molecule was counted more than once in the final tally for each chromosome. In contrast, we found that the maximum z-score for any chromosome was subtly increased from 1.9 to 2.0 when using precise counting. By performing an uncorrelated two-tailed *t*-test on the absolute values of the z-scores for all autosomes, we found that the difference between the two methods was not statistically significant (p = 0.759, n = 22×8 for each group).

### Conclusions

FAST-SeqS was capable of detecting aneuploidies in a reproducible fashion in our pilot experiments. It has advantages over unbiased whole genome sequencing in ease of implementation, cost, analysis time, and throughput. Whether it will perform as well as whole genome sequencing for fetal aneuploidy testing in the clinic can only be determined by future large-scale studies in which a large number of pregnant women are analyzed by both testing procedures.

## Supporting Information

Table S1 **Samples analyzed in this FAST-SeqS study.**
(DOC)

Table S2 **Oligonucleotides used to prepare and sequence FAST-SeqS samples.**
(DOC)

Table S3 **Sequencing characteristics of FAST-SeqS experiments.**
(DOC)
