# Supplementary material for: FAST-SeqS: A Simple and Efficient Method for the Detection of Aneuploidy by Massively Parallel Sequencing
Source: PLoS One. 2012 Jul 18;7(7):e41162. doi: 10.1371/journal.pone.0041162 (PMC3399813; doi:10.1371/journal.pone.0041162)
Supplement: Table S1 — Samples analyzed in this FAST-SeqS study. (DOC) [file pone.0041162.s001.doc]

**Table S1. Samples analyzed in this FAST-SeqS study.**

| **Individual** | **Tissue** | **Aneuploidy** | **Sample Name** |
| --- | --- | --- | --- |
| n_01 | Plasma | None | n_01_pls |
| n_02 | Plasma | None | n_02_pls |
| n_03_1 | Plasma | None | n_03_1_pls |
| n_03_2 | Plasma | None | n_03_2_pls |
| n_04 | Plasma | None | n_04_pls |
| n_05 | Plasma | None | n_05_pls |
| n_06 | Plasma | None | n_06_pls |
| n_07 | Plasma | None | n_07_pls |
| n_01 | WBC | None | n_01_wbc |
| n_02 | WBC | None | n_02_wbc |
| n_03_1 | WBC | None | n_03_1_wbc |
| n_03_2 | WBC | None | n_03_2_wbc |
| n_04 | WBC | None | n_04_wbc |
| n_05 | WBC | None | n_05_wbc |
| n_06 | WBC | None | n_06_wbc |
| n_07 | WBC | None | n_07_wbc |
| n_08 | Spleen | None | n_08 |
| n_09 | Spleen | None | n_09 |
| n_10 | Spleen | None | n_10 |
| n_11 | Spleen | None | n_11 |
| n_12 | Spleen | None | n_12 |
| n_13 | Spleen | None | n_13 |
| n_14 | Spleen | None | n_14 |
| n_15 | WBC | None | n_15 |
| t21_01 | Fibroblast | Trisomy 21 (NA02767) | t21_01 |
| t21_02 | Fibroblast | Trisomy 21 (NA04616) | t21_02 |
| t21_03 | Fibroblast | Trisomy 21 (NG05120) | t21_03 |
| t21_04 | Fibroblast | Trisomy 21 (NG05397) | t21_04 |
| t21_05 | Fibroblast | Trisomy 21 (NG07438) | t21_05 |
| t18_01 | Fibroblast | Trisomy 18 (NA03623) | t18_01 |
| t18_02 | Fibroblast | Trisomy 18 (NG12614) | t18_02 |
| t13_01 | Fibroblast | Trisomy 13 (NA03330) | t13_01 |
