# Supplementary material for: FAST-SeqS: A Simple and Efficient Method for the Detection of Aneuploidy by Massively Parallel Sequencing
Source: PLoS One. 2012 Jul 18;7(7):e41162. doi: 10.1371/journal.pone.0041162 (PMC3399813; doi:10.1371/journal.pone.0041162)
Supplement: Table S2 — Oligonucleotides used to prepare and sequence FAST-SeqS samples. (DOC) [file pone.0041162.s002.doc]

**Table S2. Oligonucleotides used to prepare and sequence FAST-SeqS samples.**

| **Legend** | | |
| --- | --- | --- |
| **FAST-1 sequence** | | |
| **Unique Identifier:**  **B, D, H, N, and V are degenerate bases according to IUPAC specifications** | | |
| **Universal primer sequence:**  **Binding site for the second round PCR amplification and universal sequencing primers** | | |
| **Index sequence:**  **Allows discrimination of samples when >1 is analyzed in the same compartment of the sequencing instrument** | | |
| **Illumina grafting sequence:**  **Facilitates hybridization to the sequencing instrument** | | |
| **Primer Name** | **Sequence (5’ to 3’)** |  |
| R1-ForA* | **CGACGTAAAACGACGGCCAGTNNNNNNNNNNNNNNNNACACAGGGAGGGGAACAT** |  |
| R1-ForB** | **CGACGTAAAACGACGGCCAGTHBVDHBVDHBVDHBVDHBVDACACAGGGAGGGGAACAT** |  |
| R1-Rev | **CACACAGGAAACAGCTATGACCATGTGCCATGGTGGTTTGCT** |  |
| R2-For | **AATGATACGGCGACCACCGAGATCTACACCGACGTAAAACGACGGCCAGT** |  |
| R2-RevA | **CAAGCAGAAGACGGCATACGAGATGATCAGCACACAGGAAACAGCTATGACCATG** |  |
| R2-RevB | **CAAGCAGAAGACGGCATACGAGATGCCAATCACACAGGAAACAGCTATGACCATG** |  |
| R2-RevC | **CAAGCAGAAGACGGCATACGAGATTGACCACACACAGGAAACAGCTATGACCATG** |  |
| R2-RevD | **CAAGCAGAAGACGGCATACGAGATTTAGGCCACACAGGAAACAGCTATGACCATG** |  |
| R2-RevE | **CAAGCAGAAGACGGCATACGAGATCTTGTACACACAGGAAACAGCTATGACCATG** |  |
| Universal Sequencing Primer | **GAGATCTACACCGACGTAAAACGACGGCCAGT** |  |
| Index Sequencing Primer | **CATGGTCATAGCTGTTTCCTGTGTG** |  |
| *****The first round primer used for all experiments except for the DNA n_08 through n_15 (see Table S1)  ******The first round primer used for experiment with DNA n_08 through n_15 (see Table S1)  **Schematic of final sequencing library (only one strand is shown, 5’ to 3’)** | | |
| **██████ – ██████ – ██████ – ████████████ –██████ –██████ – ██████** | | |
| bp: 29 21 16 or 20 ~124 – 142 25 6 24  Note: the sequences added to facilitate sequencing (i.e., sequences not in black) totaled either 121 or 125 bp, depending on which primer containing a unique identifier sequence (R1-ForA or R1-ForB) was used. | | |
