# Supplementary material for: FAST-SeqS: A Simple and Efficient Method for the Detection of Aneuploidy by Massively Parallel Sequencing
Source: PLoS One. 2012 Jul 18;7(7):e41162. doi: 10.1371/journal.pone.0041162 (PMC3399813; doi:10.1371/journal.pone.0041162)
Supplement: Table S3 — Sequencing characteristics of FAST-SeqS experiments. (DOC) [file pone.0041162.s003.doc]

**Table S3. Sequencing characteristics of FAST-SeqS experiments.**

| **Illumina Instrument** | **Sample(s) Sequenced** | **Group** | **Exp. ID** | **Samples per Lane** | **Tags Passing Chastity Filter** | **Unique Alignments** | **Unique Alignments (%)** | **Distinct Positions** |
| --- | --- | --- | --- | --- | --- | --- | --- | --- |
| HiSeq 2000 | n_01_pls | 1 | 1 | 4 | 27,179,424 | 8,303,313 | 31% | 22,589 |
| HiSeq 2000 | n_02_pls | 1 | 2 | 4 | 29,081,731 | 10,081,137 | 35% | 23,632 |
| HiSeq 2000 | n_03_1_pls | 1 | 3 | 4 | 30,074,519 | 10,205,766 | 34% | 23,608 |
| HiSeq 2000 | n_03_2_pls | 1 | 4 | 4 | 34,968,939 | 12,626,577 | 36% | 24,562 |
| HiSeq 2000 | n_04_pls | 1 | 5 | 4 | 30,842,018 | 10,492,951 | 34% | 24,163 |
| HiSeq 2000 | n_05_pls | 1 | 6 | 4 | 36,048,017 | 12,997,372 | 36% | 24,314 |
| HiSeq 2000 | n_06_pls | 1 | 7 | 4 | 32,670,848 | 11,536,414 | 35% | 23,145 |
| HiSeq 2000 | n_07_pls | 1 | 8 | 4 | 31,518,407 | 11,526,042 | 37% | 23,431 |
| HiSeq 2000 | n_01_wbc | 2 | 9 | 4 | 35,712,331 | 11,805,456 | 33% | 22,807 |
| HiSeq 2000 | n_02_wbc | 2 | 10 | 4 | 13,158,351 | 4,905,067 | 37% | 20,577 |
| HiSeq 2000 | n_03_1_wbc | 2 | 11 | 4 | 42,674,992 | 16,015,347 | 38% | 23,454 |
| HiSeq 2000 | n_03_2_wbc | 2 | 12 | 4 | 19,063,243 | 7,556,803 | 40% | 21,133 |
| HiSeq 2000 | n_04_wbc | 2 | 13 | 4 | 39,854,363 | 14,576,619 | 37% | 23,285 |
| HiSeq 2000 | n_05_wbc | 2 | 14 | 4 | 25,058,110 | 9,607,768 | 38% | 21,642 |
| HiSeq 2000 | n_06_wbc | 2 | 15 | 4 | 31,447,494 | 11,781,575 | 37% | 22,255 |
| HiSeq 2000 | n_07_wbc | 2 | 16 | 4 | 27,035,516 | 10,435,840 | 39% | 21,828 |
| GA IIx | n_08 | 3 | 17 | 2 | 11,233,449 | 4,002,305 | 36% | 20,873 |
| GA IIx | n_09 | 3 | 18 | 2 | 10,593,346 | 4,430,374 | 42% | 20,804 |
| GA IIx | n_10 | 3 | 19 | 2 | 9,015,697 | 3,909,552 | 43% | 20,392 |
| GA IIx | n_11 | 3 | 20 | 2 | 9,976,099 | 4,381,161 | 44% | 20,581 |
| GA IIx | n_12 | 3 | 21 | 2 | 9,698,517 | 4,380,884 | 45% | 20,466 |
| GA IIx | n_13 | 3 | 22 | 2 | 10,432,676 | 4,645,608 | 45% | 20,737 |
| GA IIx | n_14 | 3 | 23 | 2 | 7,871,040 | 3,514,064 | 45% | 20,135 |
| GA IIx | n_15 | 3 | 24 | 2 | 8,198,416 | 2,847,661 | 35% | 20,046 |
| GA IIx | t21_01 | 3 | 25 | 2 | 10,381,557 | 3,515,217 | 34% | 20,595 |
| GA IIx | t21_02 | 3 | 26 | 2 | 7,939,433 | 2,911,392 | 37% | 20,163 |
| GA IIx | t21_03 | 3 | 27 | 2 | 5,524,580 | 1,972,803 | 36% | 19,095 |
| GA IIx | t21_04 | 3 | 28 | 2 | 6,476,900 | 2,326,116 | 36% | 19,653 |
| GA IIx | t18_01 | 3 | 29 | 2 | 3,716,461 | 1,343,382 | 36% | 18,484 |
| GA IIx | t18_02 | 3 | 30 | 2 | 6,197,319 | 2,384,437 | 38% | 19,543 |
| GA IIx | t13_01 | 3 | 31 | 2 | 7,373,315 | 2,598,029 | 35% | 19,809 |
| GA IIx | 100% n_15 | 4 | 32 | 2 | 9,905,447 | 3,964,677 | 40% | 20,612 |
| GA IIx | 100% n_15 | 4 | 33 | 2 | 10,540,366 | 3,412,883 | 32% | 20,674 |
| GA IIx | 95% n_15 + 5% t21_05 | 4 | 34 | 2 | 10,618,558 | 4,243,752 | 40% | 21,136 |
| GA IIx | 95% n_15 + 5% t21_05 | 4 | 35 | 2 | 10,713,550 | 4,261,765 | 40% | 21,095 |
| GA IIx | 90% n_15 + 10% t21_05 | 4 | 36 | 2 | 11,170,581 | 4,453,489 | 40% | 21,030 |
| GA IIx | 75% n_15 + 25% t21_05 | 4 | 37 | 2 | 9,444,389 | 3,761,988 | 40% | 20,950 |
| HiSeq 2000 | 100% n_15 | 5 | 38 | 4 | 29,844,141 | 10,239,086 | 34% | 22,194 |
| HiSeq 2000 | 100% n_15 | 5 | 39 | 4 | 36,518,106 | 13,771,732 | 38% | 23,612 |
| HiSeq 2000 | 100% n_15 | 5 | 40 | 4 | 29,971,627 | 11,108,083 | 37% | 22,689 |
| HiSeq 2000 | 100% n_15 | 5 | 41 | 4 | 42,240,580 | 15,998,021 | 38% | 23,436 |
| HiSeq 2000 | 96% n_15 + 4% t21_05 | 5 | 42 | 4 | 22,779,370 | 7,992,518 | 35% | 21,374 |
| HiSeq 2000 | 96% n_15 + 4% t21_05 | 5 | 43 | 4 | 20,583,900 | 8,066,490 | 39% | 21,822 |
| HiSeq 2000 | 96% n_15 + 4% t21_05 | 5 | 44 | 4 | 13,435,012 | 5,239,203 | 39% | 20,555 |
| HiSeq 2000 | 96% n_15 + 4% t21_05 | 5 | 45 | 4 | 16,988,321 | 6,752,178 | 40% | 21,053 |
| HiSeq 2000 | 92% n_15 + 8% t21_05 | 5 | 46 | 4 | 31,696,544 | 10,785,467 | 34% | 22,376 |
| HiSeq 2000 | 92% n_15 + 8% t21_05 | 5 | 47 | 4 | 33,552,046 | 12,537,527 | 37% | 23,710 |
| HiSeq 2000 | 92% n_15 + 8% t21_05 | 5 | 48 | 4 | 31,650,369 | 11,635,853 | 37% | 22,784 |
| HiSeq 2000 | 92% n_15 + 8% t21_05 | 5 | 49 | 4 | 37,499,259 | 14,082,747 | 38% | 23,228 |
